# Patient perspectives and experiences with psilocybin treatment for treatment-resistant depression: a qualitative study

**DOI:** 10.1038/s41598-024-53188-9

**Published:** 2024-02-05

**Authors:** Joost J. Breeksema, Alistair Niemeijer, Erwin Krediet, Tilman Karsten, Jeanine Kamphuis, Eric Vermetten, Wim van den Brink, Robert Schoevers

**Affiliations:** 1 Department of Psychiatry, Research School of Behavioural and Cognitive Neurosciences (BCN), University of Groningen, University Medical Center Groningen, University Center of Psychiatry, Postbus 30.001, 9700 RB Groningen, The Netherlands; 2 OPEN Foundation, Amsterdam, The Netherlands; 3 Department of Care Ethics, University of Humanistic Studies, Utrecht, The Netherlands; 4 Department of Psychiatry, Leiden University Medical Center, Leiden, The Netherlands; 5 Department of Psychiatry, University of Utrecht, Utrecht, The Netherlands; 6 Academic Medical Center, Department of Psychiatry, Amsterdam UMC, Amsterdam, The Netherlands; 7 Amsterdam Neuroscience Research, Program Compulsivity, Impulsivity and Attention, Amsterdam, The Netherlands

**Keywords:** Psychology, Therapeutics

## Abstract

Psilocybin is the most researched classic psychedelic for Treatment-Resistant Depression (TRD). While optimizing set and setting are considered essential for efficacy and safety, patient perspectives on these aspects have rarely been investigated. To address this knowledge gap, the current paper explored the experiences of 11 TRD patients (8 women, 3 men) participating in a double-blind randomized clinical trial with a single session of oral (1, 10 or 25 mg) psilocybin treatment. After qualitative analysis, three major themes were identified: (1) challenges with trust-building and expectation management; (2) navigating the experience; and (3) the need for a more comprehensive treatment. Subthemes of the first theme include a general distrust in mental healthcare, trust in study therapists, limited time for preparation, and managing expectations. The second theme included the following subthemes: trusting to surrender, profound and overwhelming experiences, and music as a guide. The third theme addressed a desire for multiple psilocybin sessions, and challenges with sensemaking. Patients’ perspectives provided important insights into potential optimization of psilocybin treatment of TRD, including individualized preparation, investment in trust-building, offering additional psilocybin sessions, providing access to sustained (psycho)therapy with trusted therapists, and personalizing treatment approaches, which may also enhance real-world adaption of these treatments.

## Introduction

Depression is a common mental disorder that carries a substantial disease burden, afflicting over 300 million people worldwide^1^. Treatment of depression can be complex, and current treatment options, including various psychotherapies and antidepressant medication^2^, are not (fully) effective for up to 30% of patients^3^. Novel therapeutics like ketamine^4^ and classic psychedelics like lysergic acid diethylamide, psilocybin, ayahuasca, and dimethyltryptamine^5,6^ are increasingly investigated to treat patients with (treatment-resistant) major depressive disorder (TRD), often defined as failure to respond to two or more adequate antidepressant trials (although no consensus exists regarding amount and types of treatment)^7^.

Psilocybin is the most studied classic psychedelic: most often in relatively small samples (n = 12–59) with two recent exceptions (n = 104–233)^8,9^. In these studies, psilocybin was administered once or twice in doses ranging from 10-30 mg^8–14^. Psilocybin treatment for major depressive disorder (MDD) and TRD has recently been awarded ‘breakthrough therapy’ status by the FDA^15^. With over thirty registered trials (> 2000 patients), psilocybin treatment for depression (sometimes with comorbid anxiety, alcohol use disorder, or others) is on the forefront of the renewed interest in therapeutic use of psychedelics.

Overall, psilocybin has shown to significantly and rapidly decrease depressive symptoms with moderate effect sizes shortly after and in the week(s) following the intervention^16^. Two longer-term follow-up studies have found improvements in mood for a substantial proportion of patients six^17^ to twelve^18^ months after the last psilocybin session.

In contrast to this growing body of evidence on the clinical effects of psilocybin, including research into patients’ perspectives on these treatments is still limited. This is especially relevant when these treatments are offered to vulnerable TRD patients, who may have difficulty coping with the potentially intense or overwhelming experiences induced by psilocybin. Patients’ experiences can inform us about helpful, less helpful and negative treatment aspects, which may help optimize treatment approaches. Qualitative research is especially suitable to explore patients’ lived experiences of psychedelic sessions and on the influence of contextual factors. A few studies have employed qualitative methods to explore patient experiences with psychedelic treatments^19^, including psilocybin^20^. However, only two qualitative studies have addressed experiences with psilocybin treatment in 19 TRD patients. The first report on this study described increased connectedness and increases in acceptance as important change mechanisms^21^, whereas the second report focused exclusively on music appreciation^22^. Neither study explored other aspects of set (such as personality, expectations, intention, mood, and other psychological variables) and setting (the social, physical, and cultural environment in which the experience takes place), which are considered fundamental conditions for safe and effective psychedelic treatments^23–25^. To address this knowledge gap, the current study qualitatively explored the expectations and experiences of TRD patients who had participated in a double-blind randomized controlled dose-finding trial with a single dose of oral psilocybin (10 or 25 mg) or active placebo (1 mg).

## Results

### Themes

The following major themes were identified: (1) challenges with trust-building and expectation management; (2) navigating the experience; and (3) the need for a more comprehensive treatment. Sub-themes within the broader themes are reported below. Narrative excerpts and citations are provided throughout to illustrate participants’ perspectives.

## Challenges with trust-building and expectation management

A central theme in our analysis was the importance of developing trust and of managing expectations during the time-limited preparation phase.

### General distrust in mental healthcare

Several respondents reported difficulty trusting others in general; either as a personality characteristic or as part of their mental illness, but for some it was (also) related to a lack of trust in or disappointment with the mental healthcare system: “I must say that, honestly, I am a bit tired of therapy. I know it all, and then you get all these questions and I think: oh god here we go again. Maybe I’m a little bit therapied out.” (P7, 25 mg). Many had been involved in psychiatric care for a long time and had multiple unsuccessful treatments. They reported lacking faith in both the mental healthcare system and treatment providers, and frequently described feeling not taken seriously.

I have little illusion in [mental health treatments], let alone the antidepressants drama. You have to take them for at least six weeks, often with all the side effects, only to conclude they don’t do anything (…) The last time I thought I ought to get back on medication, I went to my psychiatrist, with whom I don’t have a click at all. Freshly graduated, not open, very reserved. (…) I just know that he is going to precisely follow guidelines of what to prescribe. (…) Like, did you become a psychiatrist to figure out via a step-by-step plan what medication I should take? Or do you also look a little further? Yeah, that disappoints me. (P8, 10 mg)

A minority also expressed distrust in psilocybin, because of the stigma associated with ‘drugs’.

I was, am, very skeptical. (…) If it sounds too good to be true, it probably is. That’s what it sounded like to me. (…) I have zero experience with drugs. (…) I don’t know what it’s going to do to me. Will I get addicted to it? (…) The [researchers] want **me** to do the study (…) [But] they don’t have to take it, it’s in their interest if I participate. (P3, 1 mg)

### Trust in study therapists

Overall, despite their general distrust and disappointment in the treatment system, most participants spoke highly of the therapists involved in the psilocybin study and felt that they had been able to establish rapport during the preparation phase. Characteristics that were explicitly valued by respondents were the sense of calm, understanding and warmth they exuded. Many reported feeling a sense of safety and confidence that they were in good hands.

One participant, who reported no discernable psychoactive effects, nevertheless stated that the study therapists had reignited her trust due to the attention and care she had received.

Everything was set up so nicely. I’m very demanding, really very perfectionistic, but I could find little that needed to be done differently. No, it was really perfect in my experience. (…) Being cared for by a professional for a whole day, they put a blanket over me, turned on music. I really felt like a child there. I could put aside all my usual guards. (…) After each hospital visit, it felt like I had been with a group of friends. (P2, 1 mg)

### Limited time for proper preparation

Many participants were anxious and nervous before their first (ever) psilocybin session. Most respondents had no prior experience with psychedelics and were unfamiliar with the experiential terrain they were about to enter. Despite their satisfaction with the trial therapists, several participants also noted that three preparatory sessions did not provide enough time to establish trust and rapport, while also discussing intentions, hopes and expectations, and preparing for what might occur during the psilocybin session. Further, participants were invited to disclose relevant (biographical or medical) background information, which they often found difficult, and which was compounded by their lack of trust in the mental health system, and the time limitations of the clinical trial.

I don’t remember to what extent we covered my, somewhat messy childhood, in detail (…) I also don’t think there was really time for that since they’re not your regular therapists. (…) In regular therapy, you just have longer conversations with someone who knows what is going on, what you are dealing with (…) Before the psilocybin, I had three moments where I talked to my therapist, and of course that also has to include the bit about how the treatment is going to go. (…) I do think there’s a certain level of trust that’s still a little bit missing in that sense. To completely surrender to it. (P9, 1 mg)

Some expressed feeling pressured to be ready for the psilocybin experience within three sessions. This feeling was enhanced by the requirement to taper off medications within the limited time frame of the study, which also led to a sense of restlessness or uneasiness. In a few cases, the uncertainty involved in participating in a clinical trial and the timing of the trial (which largely took place during the covid-19 pandemic) created further uncertainty and unrest.

I had to stop doing certain [medications] and that didn’t make me feel better, I must say. I got through it with gritted teeth, and some medication to keep me somewhat calm and to help me sleep. (…) The whole preliminary process I found very long. You just keep getting examinations and questionnaires. (P5, 10 mg)

### Managing expectations

Having been disappointed multiple times in earlier treatments, some participants tried to minimize their expectations, but others expressed hopes for a positive outcome.

I’d watched this [preparation] video of people sailing a boat on a river, experiencing the nicest things (…) [The therapists] told me that negative things could come up, but positive things as well (…) If I had something on my mind, that I could come forward, and I could work with that. (…) And yeah, that was kind of my expectation, I guess (…) But I didn’t see any cute stars or a kaleidoscope or nothing like that, and I didn’t have those feelings they had described to me (…) Just a letdown. (P5, 10 mg)

A number of participants had very specific expectations: getting insights (into their disorder, the source of their unhappiness), connecting to emotions, or being able to steer the experience. When the experience did not correspond to their expectations, they expressed feeling disappointed.

Well, the intention was to see if could see or hear something I can link to this feeling [of being very upset]. I didn’t have that at all. Just this inky black and white thing, a very intense feeling of pure darkness. (P4, 25 mg)

Several participants mentioned wishing they had been told that strong emotions may come up, and that it is okay to experience them. Some suggested using experienced patients in the preparation phase, to reverse mistrust, instill confidence, pre-empt anxiety, and/or normalize and validate their reactions to psilocybin.

If I had known that other people cried or laughed too, I wouldn’t have found it so … embarrassing. (….) I thought well, if I had known that I could have just done so, too. (P6, 10 mg)

## Navigating the experience

Navigating and surrendering to intense, profound and sometimes overwhelming experiences induced by psilocybin, sometimes guided by music, was the second major theme.

### Trusting to surrender

Participants differed substantially in their ability to let go of control. Some were able to surrender to the experience, reassured by their (trust in) therapists, but for others this remained challenging.

I didn’t feel unsafe, although I really did not feel in control of what was going on at all. (…) I guess I did feel supported at some level, because I knew there was someone I could ask to hold my hand, someone I could trust. (P12, 25 mg)

In this study, participants met the co-therapist for the first time in the last preparatory session. For some, this affected their ability to fully surrender: “Don’t just suddenly introduce a stranger into the [psilocybin room] whom you’ve never really met before.” (P6, 10 mg). The psilocybin session was mostly spent lying in bed, with two therapists continuously present. Many respondents spontaneously remarked on the awkwardness of being in this vulnerable position, which made it harder to surrender to the experience.

Two people are watching you all the time, that’s something you have to be able to put aside, this idea of: gosh, I’m being observed. Otherwise, you’re not comfortable. (P8, 10 mg).

For some, wearing an eye mask and headphones facilitated introspection and closing off from the outside world. For others, however, it induced feelings of isolation or claustrophobia, which created resistance and made giving into the experience more difficult. In others, this reinforced a pre-existing sense of disconnection or triggered traumatic memories.

[This traumatic episode] continues to haunt me today. (…) It’s still very frightening sometimes (…) I actually never shut myself off from the outside world (…) I never wear headphones or earplugs, because then I can't hear what's going on around me. [During the psilocybin session] I was cut off from everything (…) I felt trapped, because I couldn't hear and see what was happening. (P5, 10 mg)

For others, giving into the experience was challenging or even frightening due to the lack of agency or their inability to steer the experience in a specific direction, and some were unable to ask for help during difficult moments.

During preparation, you look at what you want to focus on, and then you try to kind of move towards that… But I couldn’t do anything, couldn’t steer, couldn’t get in or out, I just got dragged along, and I had no control. I wasn’t the storyteller; I was a character in a story, and I just had to wait and see what happened next. (…) That was the frustrating part. I thought, this is not what we practiced. (…) The idea was to just be aware of things and to let go. And I just couldn’t. (…) I think that if I’d had less [psilocybin] I could have steered it more. (P13, 25 mg)

### Profound and overwhelming experiences

Several participants reported intense experiences; some of which were considered positive, meaningful, or insightful.

Most of all, it was emotional (…) It was all over the place. (…) Feelings of awe. I cannot explain it. (…) Intense experiences of beauty. (…) I experienced joy at some point. Just happiness. I also experienced sadness, but not in the same way that I normally do. Not as myopic, if that makes sense. It was very deep and profound. But not necessarily negative. (P12, 25 mg)

Others reported unpleasant experiences and being overwhelmed by the intensity of these experiences, which also made it difficult for them to process these.

This overwhelming sense of deep unhappiness … was just there, suddenly. Actually, it came out of myself, but it also came over me. Everything around me, everywhere I was, the whole environment was that unhappiness, that vulnerability, that loneliness, and that being left behind. (…) [I] couldn’t do anything about it, it just came, [I] couldn’t stop it, couldn’t save [my]self. [I was] drowning in it and hoping [I would] come back up. (P13, 25 mg)

### Music as a guide

Several participants mentioned that music directed their internal experience, such that hopeful sounding music led to more hopeful experiences, with sad songs leading to feelings of sadness, and ‘burial-like’ music leading to associations with death.

At a certain point there was a bunch of heavy classical music, and then I saw a graveyard with headstones. I said: oh, there’s that grave mood again. Someone died, I just don’t know who. Like, what does it even matter. (…) And that was very liberating. Very liberating. (P7, 25 mg)

Appreciation of the fixed music playlist varied; whereas some participants did not care for the music—calling it cliché, new-agey or annoying—others found it beautiful and said it contributed positively to their experience.

It sounded extremely beautiful. Second, it made the experience more intense. (…) Another song would come on, and I would just go again 'woossssh' into the experience... So it very much guided the process. (P12, 25 mg)

## Need for a more comprehensive treatment

### Desire for multiple psilocybin sessions

Multiple participants expressed a desire for additional psilocybin sessions. A number of participants observed that getting a first (presumed) low dose had helped them become familiar with the effects of psilocybin and feel in control of the experience.

It was very nice to notice an effect but also still having some control. Even though safety was built in, I secretly found it quite nice that it wasn’t all completely … super psychedelic and out of this world. (P1, 1 mg)

Several others also mentioned that an initial lower dose would have prepared them better about what to expect in subsequent sessions, to get used to the effects of psilocybin and their own reaction to it.

I think a second time will be a bit more relaxed, [since] you know what can happen. Plus, you can build on your experiences. Of course, they can go in a different direction. So, you build on that, under the guidance of your current therapist. (…) A treatment plan should be set up around it. (…) Which means more talks, which means it’ll be more grounded too. I think that’s more constructive. You build a better foundation that way (…) It would be a shame if the treatment plan were just one session and then done. (P9, 1 mg)

Several respondents indicated that they thought one session was unlikely to have a lasting effect on their mood or symptoms and expressed a wish to integrate multiple psilocybin sessions in a therapeutic framework.

I don’t believe that one trip will completely rid me of my depression. I’m sure it requires more than that (…) Because that would make it a miracle cure [laughing] (…) I do think it’s a miraculous substance, and it might help many people. (…) But I also think that maybe it’s a process and you should do it a little more often. (P7, 25 mg)

Two participants addressed their need for additional psilocybin sessions by micro-dosing psilocybin truffles at home to sustain the initial positive effects that faded after a few weeks: *“I wanted to experience that feeling again at home.”* (P7, 25 mg).

Look, obviously after a month [the effect] is gone. And if you could get another dose the following month and a full day to surrender to it. And then again after a month and a half. Yes, then I do think that could work. (P5, 10 mg)

Neither of these participants, however, reported benefits from their microdosing sessions. One respondent mainly felt irritable and cranky, and stated that microdosing should also be done under supervision. *“It’s hard to find people who can guide you. I do miss that. It’s mainly experimenting by yourself.”* (P9, 1 mg). Another respondent stopped after three attempts, upon noticing that her depression would relapse after a few days.

### Challenges with sensemaking

Putting intense experiences into words, whether positive or negative, was a struggle for many. Some reported lacking the vocabulary, stated the experience was beyond language, or were unable to explain what had happened or how to make sense of what they had experienced.

I, um, well, it’s very hard to explain, because it was a feeling, really. I didn’t see or hear anything; it was pure feelings. (…) You can’t explain it, you really have to feel it yourself if you want to know what you’re talking about actually. But anyway, um… yes, difficult. (P4, 25 mg)

Making sense of difficult or even harrowing experiences was particularly challenging.

It had been incredibly traumatizing … it was almost as if I had a new trauma purely from that treatment because I had been so miserable and so lonely and sad. Eight hours of crying (…) It was such an intense experience with so many emotions. (…) For two, three months, every time I talked about it, I burst into tears. I would get panicky, start having panic attacks and dreaming about it (…) It’s slowly ebbing away now, but in the beginning, it was so intense I didn’t know what to do with it. (P13, 25 mg)

Most participants expressed that processing, integrating or being able to discuss experiences required time to understand their meaning or significance. Relevant insights sometimes became apparent over the days after the psilocybin session, but occasionally this took weeks or even months, which also led to a need for additional support.

I did expect a little more support. And I really did feel the need for it (…) There were two talks and that was it. But I did feel like I needed more, and it wasn’t possible. To me it felt like: this is it, bye-bye, you’re on your own. (…) And I didn’t have a therapist at that time, so I couldn’t rely on that either. (P5, 10 mg)

For some, integration sessions were useful, but others thought that integration sessions were mainly meant to talk about the experiences itself but did not assist with sensemaking in the context of their life and disorder. Some participants stated that a framework, which should involve therapy, meaning making and a supportive environment, would have been helpful with making changes and integrating experiences into daily life.

I have this experience, and I have talked about it two or three times, but I have no idea what to do with it now. Yes, there are things that I can work on myself, but I don’t know how to do that, I don’t know how I can learn to not? let things slip away from me, I don’t know how to get less emotional about everything and everyone. (P13, 25 mg)

For some participants, the weekly phone questionnaires that were part of the study design felt burdensome and repetitive, and contrasted negatively with their need to discuss and process their experiences with a trusted therapist. The protocol included two post-psilocybin talk sessions, the first of which was planned on the day after the psilocybin session, the second a week later. For some participants these came too soon, as they were not yet ready or able to talk about their experiences.

Right after, I just couldn’t talk about it yet (…) The wound was still wide open. Let’s just say it was too painful to start talking about it at that time. (P13, 25 mg)

For others, this was the opposite; they noted a missed opportunity to capitalize on their temporarily suspended anxiety, lack of self-criticism or increased openness in a more immediate timeframe.

Right after, I didn’t hate myself so much (…) It was as if someone had turned on the light. (…) And I wasn't afraid of other people because you cannot be afraid of something that is a part of you (…) But it was short-lived, and now I think those insights are all nonsense. I hate myself again. (…) But when I didn’t hate myself, I thought: Oh! Now I know what I have to do to solve all this. But yeah, that’s all gone again now. (…) If I could have talked about everything to someone I trusted right at that moment, I think a lot would have been resolved. But now: nothing at all. (P6, 10 mg)

Finally, multiple respondents suggested that connecting with other participants throughout the process could help create a sense of connection, help with validation and feeling understood.

When I’m with folks who have the same quirks as me, I do feel part of a group. It’s just really nice not to feel like an outsider. (…) And if I were part of a psilocybin group (…) I would want to know what someone else’s experience was like. So I can say: yes, I saw that too. Or I have also experienced that. And that might make me start reflecting again. (P5, 10 mg)

## Discussion

The present qualitative study identified several elements that affected patients’ appreciation of a psilocybin treatment for TRD in a clinical trial context. The first major theme revolved around challenges with trust-building and expectation-management, which included general mistrust in mental healthcare, trust in study therapists, time pressure to be properly prepared, and the importance of managing expectations. A second major theme dealt with surrender to and navigating (challenging) experiences, and the role of music. The final theme was patients’ expressed desire for a more comprehensive treatment, which includes multiple, dose-escalated, psilocybin sessions, and challenges with sensemaking. This qualitative data adds an important personal perspective to the published research on psilocybin safety and efficacy. Patient experiences of psilocybin treatment (context) are sparse^20^. The personal accounts documented in this study vividly illustrate the importance of substance (psilocybin, dose), set (expectations, preparation, trust) and setting (the physical and interpersonal treatment context, music) for treatment experience and outcome, concepts already formulated in the early 1960s^23,24,26,27^, and offer suggestions to optimize treatment approaches in future naturalistic studies and in standard care.

### Trust

It is important to recognize that patients with treatment resistant mental disorders often have a long history in the mental healthcare system with limited success, and as a result, may have become increasingly skeptical and distrustful of psychiatric treatment in general^28^. Such feelings may complicate building a trusting therapeutic relationship with new therapists. Our findings indicate that this is feasible, even within the time-limited context of an RCT (although meeting the co-therapist only once before the psilocybin session was suboptimal in some cases). Trust is a crucial ingredient in human relationships^29^ and trust and therapeutic alliance are key factors underlying change processes in regular^30^ and psychedelic psychotherapy^31–33^, which patients affirm^19,21,34^. This was elegantly formulated by the pioneering psychedelic psychotherapist Stanislav Grof: “*A good therapeutic relationship helps the patient to let go of psychological defenses, surrender to the experience, and endure the difficult periods of sessions characterized by intense physical and emotional suffering or confusion*”^35^.

### The (in)ability to surrender

As we illustrated in another qualitative study, the (in)ability to surrender and let go of control was a central concern for TRD patients treated with oral esketamine^36^; this theme also recurred in the current study on psilocybin treatment. In several prospective studies on psychedelic use, a state of surrender before ingestion^37^, ‘feeling ready’ and ‘having clear intentions’^38^ were key predictors of positive outcomes, whereas anxiety, nervousness and preoccupation predicted adverse reactions (37,39,40). This can be specifically challenging in patients with (severe) mental disorders, who have often had negative early life experiences. Having faced childhood adversity is not only strongly associated with the onset of, and a more severe course of, psychopathology (including TRD)^41–44^, it may also hinder forming secure attachments and complicate the establishment of trust or interpersonal rapport^45^. This constellation can in turn make it more difficult for patients to let go. Patients with a range of psychiatric disorders including depression also typically score high on the personality trait of neuroticism^46,47^, which is associated with increased adverse reactions to psychedelics and likely moderated by an inability to ‘let go’ or minimize negative reactivity to difficult experiences^48^. Interestingly, the mantra ‘trust, let go, be open’ that is sometimes offered as a reminder to help people cope with difficult psychedelic experiences^49^ refers to competencies that TRD patients already struggle with, emphasizing the importance of offering additional support and more extensive preparation for patients with (severe) mental disorders.

### Preparation, education, expectation

While building rapport with therapists seemed feasible during preparation sessions, during this time-limited phase participants were also asked to disclose relevant personal information; to be educated about the range of potential effects of psilocybin; familiarize themselves with the (interpersonal) context of the psilocybin session; and finally, to discuss hopes and expectations. The latter is particularly important in light of the widespread, positive media coverage on psychedelics (the ‘Pollan Effect’)^50^. This expectancy effect not only represents a significant methodological challenge for clinical research^51,52^, it can also inflate patients’ expectations. More time may be needed for psychoeducation, to explicitly address unrealistic expectations, mitigate false hopes, and coping with potential disappointment after receiving a low or placebo dose. Future designs should also reconsider the need to discontinue the prescription of serotonergic antidepressants before initiating psilocybin therapy, which was difficult for several patients in our study. A post hoc analysis of a one recent trial^12^ indicated that patients who received psilocybin without discontinuation of their antidepressants had better outcomes than those who did^53^. Two small-scale trials—one in healthy participants pre-dosed with the SSRI escitalopram^54^ and one in TRD patients receiving psilocybin adjunctive to an SSRI^55^—also indicate that subjective effects or therapeutic outcomes are not attenuated, suggesting that co-administration is no problem, warranting a careful evaluation of the requirement to discontinue antidepressants prior to psilocybin administration.

Participants are likely to benefit from more tailored instructions and practical strategies related to being (un)able to direct experiences; handling loss of control; the potential awkwardness of spending eight hours supine being observed by two therapists; and coping with overwhelming, potentially troubling experiences. Employing (former) patients with experience in psychedelic treatments, e.g. by providing peer education in the preparation phase, was suggested as a possibility to help normalize and validate patients’ reactions to such experiences.

### Customizing psilocybin treatment

The degree to which some patients were overwhelmed by dark and despairing experiences raises the important question whether it is ethical to expose psychologically vulnerable patients to situations that may induce additional suffering. Challenging experiences have been described in other qualitative studies on psilocybin treatment too^21,56–58^ albeit often in the context of eventual (meaningful) resolution. Our study suggests that for some patients these painful experiences do not always lead to transformation or positive outcomes. Whether this means that these treatments are simply not suitable for all TRD patients, or whether these concerns can be addressed by optimizing dose and treatment (context) is an important question that warrants serious consideration when these treatments become registered. Future studies should carefully scrutinize challenging experiences and their relation to personality characteristics and psychiatric history, in order to refine eligibility criteria for participation in psychedelic-assisted treatments. As we reported elsewhere, adverse events are often registered inadequately and inconsistently, and our understanding of difficult emotional experiences, often categorized as ‘anxiety’, needs to be improved in the context of psychedelic treatment^59^. This is especially salient as cathartic experiences or emotional breakthroughs, as well as decreased thought suppression^60^, may be both challenging as well as important therapeutic mechanisms for some patients^19,61^. This tenuous balance between distressing and therapeutic effects is likely dependent on the complex interaction of patient characteristics, therapeutic frameworks, expectancy, readiness, ability to surrender to the experience, and the availability of support with integration.

Based on patients’ reports, future approaches should consider gradually increasing intensity over more than one session, starting with doses that allow some measure of control and that enable patients to become familiar with the experiential terrain, as well as to help them make sense of their experiences. Offering additional psilocybin sessions is also important in light of the availability of psychedelics outside clinical settings; in fact, two participants in this study, as has been reported in another study^21^, self-experimented with (subperceptual doses of) psilocybin truffles, which in the absence of adequate support or therapy may worsen symptoms, destabilize patients, or cause harm. While a number of survey studies indicate potential (self-reported) positive effects on mental health and mood, respondents have also reported adverse effects^62–65^. These effects have not been studied in TRD patients, except for one case report^66^, and there is evidence to suggest that set and setting variables influence outcomes even when taking subperceptual doses^67,68^.

Of the participants in this study, there were no obvious relations between the content and valence of their experiences, their ability to recall or verbalize their experiences, treatment outcomes, and dose. Three out of the 11 participants in the present study were responders and in remission at week 12. Interestingly, two had received 1 mg and one had received the full dose of 25 mg. In fact, one participant (P3, 1 mg) slept throughout most of his session and was completely symptom free at the time of interview (8 months later), whereas another participant (P12, 25 mg) had deeply meaningful (albeit fairly ineffable) experiences and was also in remission months later. The remaining 8 participants had no statistically significant changes in MADRS score. It should be noted that this is not reflective of the participants in the main trial, in which 20% in the 25 mg group were responders at week 12, versus 5% in the 10 mg group and 10% in the 1 mg group^8^.

Our results show variation in patient needs regarding timing, amount, objectives, and structure of the psilocybin and (integrative) therapy sessions. Clearly, these cannot all be accommodated in the context of an RCT, which requires a rigid structure to obtain evidence about efficacy and demonstrate internal validity in a placebo-controlled design, at the likely cost of external or ecological validity^69^, and necessarily limiting the possibility of tailoring the treatment to individual patients’ needs. With the increasing recognition that best practices around therapeutic applications of psychedelics need to be established^70^, our findings argue that this may include a more flexible, personalized approach that considers patients’ personal situations and needs rather than a fixed timing and number of therapy sessions. In addition, regular treatment could consider involving patients’ own (psycho)therapists in the process, as they are already familiar with patients’ personality, psychological makeup, and biographical background. This might entail first educating these therapists about the psilocybin treatment, but their involvement can help facilitate the interpretation of experiences in the context of patients’ life histories. Finally, highly structured clinical trial parameters and limited therapeutic flexibility means that naturalistic follow-up studies are needed to fully establish the ‘real life’ potential of these treatments.

Many of the above findings are in line with a recent paper which conceptualizes patient readiness, suggesting that it can be improved by optimizing therapeutic alliance, patient presentation, safety, and by personalizing treatment approaches^71^. Based on the insights attained in this qualitative study, several recommendations have been collected in Table 1.

**Table 1:** Recommendations to improve future clinical trial and treatment designs.

*All phases*	
Educate and involve regular therapists	Educate and involve regular therapists throughout the treatment, if possible, to help with sensemaking and integration afterwards
Balance time requirements in trials	Participant time investment in clinical trials may be experienced as burdensome; it is worth considering offering equal amounts of time spent with therapists in return
*Preparatory phase*	
Developing trust with all therapists	Allow for enough time for patients to build trust with all therapists present during sessions; some patients may need more time than others
Expectation management	Explicitly address any hopes and expectations and allow for the possibility of disappointment, also considering the potentially of low/placebo doses
Practice with surrender	Provide concrete and practical instructions that can help patients to give into (challenging) experiences during psilocybin sessions
Employ experienced experts	Employing (former) patients with experience in psychedelic treatments beforehand, e.g. in peer-to-peer education, may help in normalizing and validating experiences
*Psilocybin session phase*	
Explore tailored music playlists	Music is strongly tied to personal preference. It is worth investigating whether personally optimized music can facilitate surrender
Offer multiple (repeat) sessions	Multiple psilocybin sessions may be needed to achieve sustained improvements in therapeutic outcomes and quality of life
Gradually build up dosing	Starting with low(er) doses allows patients to be accustomed to the altered states of consciousness
*Post-session/integration phase*	
Customize integrative therapy	Where possible, personalize the amount, type, and timing of integrative (psycho)therapy as related to patient characteristics and situations
Sustained therapeutic support	Handling and integrating intense experiences may require more time than study contexts allow for; facilitating community support groups may (partially) compensate for this

### Limitations

A first limitation of this qualitative study is the fact that both researchers and participants were blinded to treatment condition and to the treatment outcome at the time of interview and data analysis, resulting in a blind assessment of a heterogenous group. On the other hand, this also led to a level of variation, detail and richness in participants’ accounts. A second limitation is that the interviews were conducted at the peak of the Covid-19 pandemic, severely restricting the possibility for in-person interviews; video conferencing software was used instead. This presented marginal technical difficulties and potentially affected the interviewer’s ability to establish rapport and pick up on non-verbal and contextual clues. Third, in order not to interfere with clinical trial requirements, interviews could only be conducted after conclusion of the long-term follow-up session. In the 3 to 15 months after the psilocybin session, participants may have had other experiences and experienced changes in their mental status that may have affected their outlook and interpretation of this treatment and may have hindered recall and detailed descriptions of their experiences. A fourth potential limitation is that participants were invited by study coordinators from two study sites in one country, which may not be representative for all sites in this international multi-center clinical trial. Although patients, therapists, and researchers were all still blinded during the recruitment phase, inviting participants based on their perceived burden capacity may have introduced a sampling bias. At the same time, participants with both positive and negative experiences were included in this study, which reduces the likelihood of (non)response bias. Further, patient demographics and therapist experience and competencies may have varied between sites, which may limit generalizability of these findings. Finally, as mentioned before, our interviews reflect the fact that psilocybin treatment took place in the context of a clinical trial, and some of the complexities highlighted by participants might be specifically related to the restricted clinical trial context and do not necessarily translate to future, more flexible real life treatment settings.

## Conclusion

Future approaches investigating or implementing psilocybin treatment for patients with TRD should carefully balance the time investment required by patients with the time investment that can be offered by therapists/researchers. To facilitate surrender to the experience, and sense-making of intense and overwhelming experiences afterwards, it is imperative to invest in building trust, customizing education, and managing expectations. Gradually increasing the intensity of psilocybin experiences over the course of multiple sessions can help familiarize patients with altered states of consciousness. This can empower them and assist with letting go of control during sessions. Access to sustained (psycho)therapy, possibly via patients’ own regular therapists, should ideally be integrated in the treatment. Involving experienced patients in both trial and intervention design, and in the therapeutic process (through peer or community support in preparation and integration) can assist in patient retention, prevent dropout due to overburdening, and most importantly, help improve therapeutic outcomes. Finally, in addition to highly needed efficacy studies we call for additional naturalistic studies that allow for more tailored therapeutic approaches, to establish the real-life potential of psilocybin in the treatment of patients with mental disorders.

## Methods

### Design

This qualitative study was designed using an Interpretative Phenomenological Analysis (IPA) data collection and analysis framework^72,73^, conducting individual in-depth interviews to obtain detailed descriptions of participants’ experiences. The main aim was to understand how patients experienced the psilocybin treatment, and to describe their lived experiences, which is a core element of IPA^73–75^.

### Treatment setting and study participants

We used purposive sampling to recruit patients who had participated in a phase 2b double-blind trial into psilocybin for TRD. All participants were adults with a single or recurrent episode of MDD without psychotic features who met TRD criteria, defined as failure to respond to at least two antidepressants with adequate doses and durations. Participants were required to discontinue their current antidepressant and other contra-indicated medications at least 2 weeks prior to the psilocybin dosing day and were requested to remain off antidepressants at least until the primary end point of the study (3 weeks after the psilocybin session) and during the 12 weeks after the dosing day.

All participants had three 60–90-min preparatory sessions prior to the psilocybin session: all three sessions with their lead therapist, the third session also included a co-therapist. Both therapists were present during the psilocybin session and the next-day integration session; a second integration session, one-week post-psilocybin, was conducted only with the lead therapist. Participants were randomized to receive a single dose of either 1 mg (as an active control), 10 mg, or 25 mg of a proprietary, synthetic formulation of psilocybin. Further details of the study are published elsewhere^8^. All participants, therapists, and investigators were blind to the treatment condition at randomization, as well as during the qualitative interview and data analysis described in this report.

Between November 2019 and October 2021, study coordinators at two study sites (University Medical Centers at Groningen and Utrecht, the Netherlands) approached participants after having completed the final RCT follow-up measurements. Selection was based on clinical assessment of patient suitability by study coordinators and principal investigators, i.e., patients that were likely to be willing to participate and for whom participation would not be excessively burdensome. Study drop-outs were not approached. Of all 44 participants at these two sites, 22 were approached and of these 13 agreed to participate in this qualitative study, but two did not show up. The remaining 11 participants (8 women, 3 men) provided written informed consent and participated in a single, semi-structured interview. Participant ages ranged from 24 to 66 (average 45.5) years. After de-blinding, 4 participants turned out to be randomized in the 1 mg control condition; 3 received 10 mg; and 4 had received the highest dose (25 mg) of psilocybin. An overview of basic demographic and response information can be found in Table 2.

**Table 2:** Participant demographics and depression scores.

Participant	Age	Sex	Psilocybin dose^‡^	MADRS total score			Time of interview since psilocybin (months)
				Baseline	Week 3	Week 12	
P1*^†§^	53	M	1 mg	34	16	3	10m
P2	59	F	1 mg	20	50	41	7m
P3*^†§^	50	M	1 mg	30	5	0	8m
P4	32	F	25 mg	29	19	15	10m
P5*	44	M	10 mg	30	15	25	6m
P6	45	F	10 mg	41	36	37	4m
P7	64	F	25 mg	21	21	22	15m
P8	66	F	10 mg	30	29	19	5m
P9	29	F	1 mg	28	29	22	3m
P12*^†§^	34	F	25 mg	29	7	4	3m
P13	24	F	25 mg	34	32	33	4m

*Response at week 3, defined as a decrease of at least 50% from baseline in the Montgomery–Åsberg Depression Rating Scale (MADRS) total score.

^†^Sustained response at week 12, defined as a week 3 response sustained through week 12.

^§^Remission, defined as an MADRS total score of 10 or less.

^‡^Both psilocybin dose and response status were obtained after data collection and analysis.

### Data collection and analysis

Interviews were conducted between 3 and 15 months (average: 6.5 months) after the psilocybin session. All interviews were conducted by the first author, who was not involved in the trial nor had prior contact with participants. Interviews loosely followed an interview guide, based on a previous study in which we explored patients perspectives on oral esketamine treatment for TRD^36^. The interview guide included open-ended questions, e.g., ‘what were your expectations of this treatment?’, ‘how did you experience the preparation sessions?’, or ‘can you describe what happened during the psilocybin session?’, intended to gauge patients’ impressions of the treatment process, to understand how patients made sense of their experiences, and to explore factors that may have benefitted or negatively impacted the experience. See Appendix 1 for the full interview guide. Average interview duration was 75 min (range 41–120 min). Due to Covid-19 related restrictions, all interviews were conducted via videoconferencing software and audio-recorded. Analysis of interview transcripts was conducted with the aid of MAXQDA, following IPA directions^72,73,76^. Emergent themes were discussed within the multidisciplinary research team. For a more detailed description of the analysis procedure and steps, please see^36^. The Standards for Reporting Qualitative Research (SRQR) and the 32-item COREQ (consolidated criteria for reporting qualitative research) checklist were used to ensure methodological rigor^77,78^.

### Ethics statement

The qualitative study protocol (Ref. M20.256068/METc 2020/352) was reviewed by the Medical Ethics Review Board of the University Medical Center Groningen (METc UMCG), which exempted the protocols from ethical review, concluding that these do not constitute clinical research with human subjects as meant under the Dutch Medical Research involving Human Subjects Act (WMO) 1999, and that approval was therefore not needed. All procedures were performed in accordance with the Declaration of Helsinki. All participants provided written consent, after being informed about the voluntary nature of their participation, the possibility to withdraw at any point, and guarantees of confidentiality and anonymity. No compensation was offered. Audio recordings and demographic information of participants were stored on separate secure servers.

### Supplementary Information

Supplementary Information.

## Data Availability

The data that support the findings of this study are not publicly available due to reasons of privacy of the respondents, and data protection regulations. Data may be made accessible upon request and after consultation with the data managing officers from the University Medical Center Groningen or by contacting the corresponding author.
