# Supplementary material for: Patient perspectives and experiences with psilocybin treatment for treatment-resistant depression: a qualitative study
Source: Sci Rep. 2024 Feb 5;14:2929. doi: 10.1038/s41598-024-53188-9 (PMC10844281; doi:10.1038/s41598-024-53188-9)
Supplement: Supplementary file 1 — Supplementary Information. [file 41598_2024_53188_MOESM1_ESM.docx]

**Interview guide psilocybin treatment for TRD**

- To begin with, can you please briefly share something about yourself?
- How did you come to participate in this psilocybin trial?
  - *Prompt: what was the trigger/what were your reasons for participating?*

**Research expectations**

- Can you describe how you felt before participating in this study?
  - *Prompt: What symptoms, what were you most bothered by, what did you want to work on?*
- What were your expectations of this study/treatment?
  - *prompt: What did you hope to get out of this treatment? themes, topics, symptoms, feelings*
- What issues did you expect might surface during the treatment?
- How did you experience the preparation sessions and your practitioner beforehand?

**Psilocybin session itself**

- Can you describe what happened during the psilocybin session? Can you describe what happened during the psilocybin sessions?
  - What sensory changes did you experience (seen, felt, heard)?
  - What inner changes have you experienced?
  - What insights or new insights have you gained?
  - What emotions came up during your experiences?
  - What memories surfaced during your experience?
- What significance did these experiences have for you?
- What was your experience with the treatment room and the two therapists present during the psilocybin session?
  - *Prompt: safety, treatment room, preparation, emotional safety, therapists, both positive and negative*
- Can you comment on how these affected your experience during the psilocybin session?

**Integration**

- How did you feel immediately after the session ended? And the next day/days/weeks after?
  - *Prompt: did you feel better, worse, calmer, more restless?*
- Can you share a little about what it's like to make sense of the experiences from the psilocybin sessions, and how you make these a part of your daily life?
- What is it like to talk to others about your experiences with this treatment?
  - *Prompt: partners, family, friends, colleagues*

**Effect of treatment**

- How can you tell whether the treatment has been effective?
  - *Prompt: can you say anything about the impact of the treatment? only positive or negative? What do partners/friends/family say? [sleep/nightmares, emotions, meaningfulness, creativity, relationships family/friends, work, hobbies, drinking, medications, drug use, nature, activities]*
- Looking at yourself, what do you see as the most significant changes compared to before this study began?
- Can you explain in your own words how you think this treatment works?
- If you had the opportunity to change something about the treatment, what would you do differently?
  - *Prompt: would you recommend this treatment to others who are in a similar position?*
- Is there anything else you would like to share?
